# Supplementary material for: Elite sport hubs during COVID-19: The job demands and resources that exist for athletes
Source: PLoS One. 2022 Jul 5;17(7):e0269817. doi: 10.1371/journal.pone.0269817 (PMC9255745; doi:10.1371/journal.pone.0269817)
Supplement: S1 Table — Main concepts and example questions for each interview. (DOCX) [file pone.0269817.s001.docx]

**S1 Table. Interview Guide**

| **Interview Guide**  Four main concepts will be covered in each interview. |
| --- |
| **1.**     **Player Wellbeing**    Example Questions  ·       Tell me about your time so far in the hub.  ·       Follow up:  o    Are you enjoying your time in the hub?  o    Tell me about the structure of your typical day.  o    How do you feel about that?  o    Has your social media usage increased? |
| **2.**     **Player Performance**    Example Questions  ·       How are you finding the condensed season?  ·       Follow Up:  o    How do you feel during training?  o    How have you been feeling on the court?  o    Do you notice any difference on game day to how you felt during previous competitions? |
| **3.**     **Support Systems**    Example Questions  ·       Do you have easy access to any support services you might need?  ·       Follow up:  o    Have you been accessing more support services than in the past?  o    Is there a support service you have found particularly helpful during the hub?  o    Are there any services you wish you had better access to? |
| **4.**     **Logistics**    Example Questions  ·       Tell me about the impact of the hub on your life outside of playing basketball, like other work, education or family?  ·       Follow up:  o    How do you feel about that?  o    Has your work load increased or decreased? (if applicable) |
