# Supplementary material for: Elite sport hubs during COVID-19: The job demands and resources that exist for athletes
Source: PLoS One. 2022 Jul 5;17(7):e0269817. doi: 10.1371/journal.pone.0269817 (PMC9255745; doi:10.1371/journal.pone.0269817)
Supplement: S2 Table — Nodes and examples of sub-nodes that were used to code the qualitative data. (DOCX) [file pone.0269817.s002.docx]

**S2 Table. Coding Structure**

| **Node** | **Sub-Node Examples** |
| --- | --- |
| **Job Demands** | Complex or boring work |
|  | Emotionally draining work |
|  | Unpleasant or stressful working environment |
| **Job Resources** | Autonomy or control over work |
|  | Family or external support |
|  | Mentoring |
|  | Physical recovery provisions and facilities |
| **Basketball Logistics** | Accommodation |
|  | Food |
|  | Recovery and sleep |
| **Coaching** | Positive/negative coaching experiences |
| **Friends and Family** |  |
| **Work and Study** |  |
| **Support Services** | Mental health |
|  | Nutrition and hydration |
|  | Physical recovery services |
